# Determination of the average crystallite size and the crystallite size distribution: the envelope function approach EnvACS

**DOI:** 10.1107/S1600576724007362

**Published:** 2024-09-17

**Authors:** Thorsten M. Gesing, Lars Robben

**Affiliations:** a Institute of Inorganic Chemistry and Crystallography University of Bremen 28359Leobener Strasse 7 Bremen Germany; b MAPEX Center for Materials and Processes University of Bremen Bibliothekstrasse 1 28359Bremen Germany; The University of Western Australia, Australia

**Keywords:** average crystallite size, Scherrer equation, pair distribution functions

## Abstract

A procedure is presented to exactly obtain the apparent average crystallite size (ACS) of powder samples using standard in-house powder diffraction experiments without any restriction originating from the Scherrer equation. Additionally, the crystallite size distribution within the sample can be evaluated. To achieve this, powder diffractograms are background corrected and long-range radial distribution functions *G*(*r*) up to 300 nm are calculated from the diffraction data. The envelope function *f*^env^ of *G*(*r*) is approximated by a procedure determining the absolute maxima of *G*(*r*) in a certain interval (*r* range). Fitting of an ACS distribution envelope function to this approximation gives the ACS and its distribution. The method is tested on diffractograms of LaB_6_ standard reference materials measured with different wavelengths to demonstrate the validity of the approach and to clarify the influence of the wavelength used. The latter results in a general description of the maximum observable average crystallite size, which depends on the instrument and wavelength used. The crystallite site distribution is compared with particle size distributions based on transmission electron microscopy investigations, providing an approximation of the average number of crystallites per particle.

## Abstract 2

A procedure to determine the average crystallite size and its distribution using the radial distribution function *G*(*r*) of long-wavelength diffraction experiments is presented and tested.

## 1. Introduction

**Figure 1:**
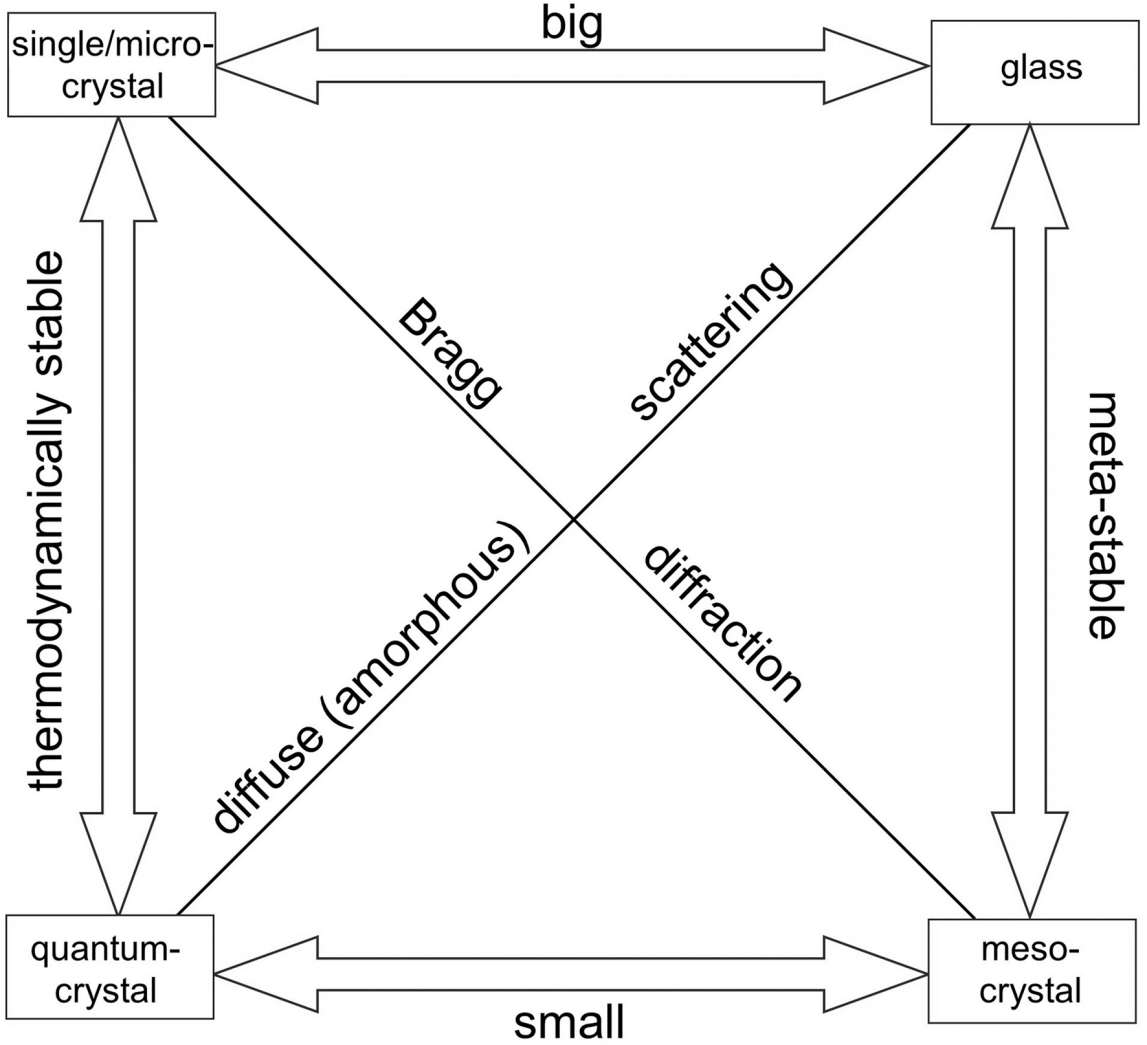
Greimas semiotic square of atomic arrangements, with the pairs of opposites thermodynamically stable (left-hand side) versus metastable (right-hand side) and big (micrometres, top) versus small (nanoscale, bottom), within X-ray diffraction.

The determination of average crystallite sizes of synthesized samples is an important tool to understand the physical properties of materials, especially if their dimension deviates from the microcrystalline state. The microcrystalline state corresponds to bulk material, and it shifts to the nano- or the quantum-crystalline state (Gesing *et al.*, 2022 ▸) if the average crystallite size is reduced and surface properties are of increasing importance. Micro-, nano- and quantum-crystallites can be obtained as thermodynamically stable phases ranging from bulk- to surface-dominated, whereas metastable phases can be obtained at larger dimensions as glasses (Warren, 1934 ▸) or very small crystallites, which are obtained as meso-crystals (Song & Cölfen, 2010 ▸). From the diffraction point of view, quantum-crystalline and glassy materials are termed (X-ray) amorphous, as pointed out in Fig. 1 ▸, and their diffraction patterns do not allow a distinction between these two states. On the other hand, micro- and meso-crystalline phases show distinct Bragg diffraction (Friedrich *et al.*, 1912 ▸; Bragg & Bragg, 1913 ▸).

Concerning the average crystallite size and the possibility of its determination using diffraction experiments, Max von Laue wrote in 1926 (von Laue, 1926 ▸), ‘*Es dürfte danach die Möglichkeit gegeben sein, aus der Breite der Interferenzringe die allgemeine Gestalt der Kristallteilchen zu ermitteln.*’ The translation of this sentence is, ‘*It should then be possible to determine the general shape of the crystal particles from the width of the interference rings.*’ This introduces the expression ‘crystalline particle’ which must be defined to avoid nowadays a general confusion about terms.

Size determination from diffraction data is only possible for a single crystallite; a powder wide-angle X-ray scattering experiment averages over all available crystallites, with all their different morphologies, leading to an apparent average crystallite size (ACS). The diffraction data therefore also contain information about the distribution of these crystallites. If several of these crystallites are agglomerated, being either crystalline or amorphous, a particle is formed which could well be determined using investigations like diffuse light scattering (DLS), but not via the broadening (von Laue, 1926 ▸) of Bragg reflections (Bragg & Bragg, 1913 ▸). The broadening effect was approximated (Patterson, 1939 ▸) by Scherrer (1918 ▸) to be
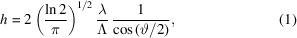
where *h* is the half-width of the reflection (denoted *B* in the following text) and ϑ is the scattering angle (we will use θ in the following text). 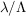 is the ratio of the monochromatic X-ray radiation wavelength (λ) and the edge length of the crystallite, which is assumed to be in the form of a cube (hereinafter Λ will be replaced by ɛ), resulting in
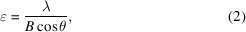
with *B* the integral breadth of the reflection (containing contributions from the sample and the instrument used), λ the wavelength and ɛ the size of the crystallites.

The constant 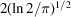 ≃ 0.9394 introduced by Scherrer is an approximated correction term for cube-shaped crystals, assuming all of them to be equivalent in size (no size distribution). That means that the apparent average crystallite size *L* relates to the true size ɛ via a constant *K* (Langford & Wilson, 1978 ▸):
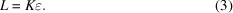


Combining equations (2) and (3) and considering only the reflection broadening caused by the ACS, denoted *b*, the resulting equation is known as the Scherrer equation, which is valid for a number of lattice planes in one direction and is given in the form (Patterson, 1939 ▸; Guinier, 1963 ▸)
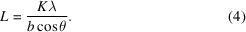
*K* is often simply assumed to be 0.94 for cube-shaped crystals and 0.89 for plate-like crystals, or even 0.9 for a general case (Guinier, 1963 ▸). Nonetheless, *K* depends on the way the reflection breadths are determined, on the shape of the crystallites and on the distribution of crystallite sizes (Friedrich *et al.*, 1912 ▸). For example, the Miller indices of the diffraction peaks must be considered in such a way that, depending on the compound’s symmetry and the crystallites’ shapes, different values of *K* must be applied to the different diffraction peaks (Langford & Wilson, 1978 ▸). Additionally, the measured material-dependent reflection broadening *B* must be reduced by the instrumental contribution β.

Guinier (1963 ▸) pointed out that the crystallite broadening *b* could be much better calculated from the measured reflection broadening *B* using
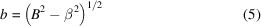
instead of a simple subtraction
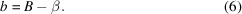
Additionally, it is necessary that ‘*in any case the correction must be small if the result is to be valid*’ (Guinier, 1963 ▸). That means that, using the Scherrer equation, the ACS must be very small compared with the standard material used for the determination of the instrumental broadening.

These points make the ‘stand-alone’ application of the Scherrer equation a complicated endeavor if all possible influences are considered in the calculation of the crystallite sizes. Besides these influences, the strain broadening of the reflections also plays an important role in many samples. To separate these two mechanisms, methods like Williamson–Hall plots (Hall & Williamson, 1951*a* ▸,*b* ▸) or those of Warren & Averbach (1952 ▸) have been applied to scattering data. Such methods were developed to evaluate the internal structure changes in *e.g.* cold-rolled metals, using all the boundary conditions the materials impress on the scattering data, but not the crystallite sizes of nanomaterial powders.

Ultimately, the correct determination of the average crystallite size using the Scherrer equation directly is not that easy and in a lot of cases diffraction data may not be good enough to allow determination beyond the assumption of spherical crystallites. The convolution of peak broadening functions based on the Scherrer equation with a calculated diffraction pattern, as is realized in the common Rietveld programs, is a much better approach in this respect, because it considers at least the diffraction angle-dependent instrumental broadening. However, Scardi & Leoni (2006 ▸) emphasized that Voigt profiles are ‘*not suitable to model the diffraction patterns of lognormal dispersed nanocrystalline materials*’ and that an ‘*a priori chosen profile functions can hardly adapt to profiles by any possible combination of line broadening sources*’. Nevertheless, the correct reflection treatment concerning *K* is still not included and the evaluated ACS is still an estimation.

Considering the realm of nano-crystallites, Weidenthaler (2011 ▸) collected common mistakes and pitfalls and pointed out that only a combined approach with several methods is able to obtain reliable information on the shape of nano­materials. However, for nanomaterials a different approach is often much better suited, because the diffraction peak breadth increases so much that on one hand the instrumental broadening corrections do not contribute much to the total peak broadening, and on the other hand it becomes more and more difficult to distinguish the sample’s diffraction from the noise of the measurement (Krämer *et al.*, 2023 ▸). Additionally, due to the increasing influence of the surface atoms (Gesing *et al.*, 2022 ▸) changes from the bulk structure are obtained (Kirsch *et al.*, 2019 ▸). Thus, structure examinations of nanomaterials with scattering methods are better carried out in real space using the pair distribution function (PDF) calculated from reciprocal-space scattering data. For this approach short wavelength radiation is usually used to obtain a high absolute value of the scattering vector *Q* [*Q* = (4π/λ) sin θ, where θ is half the scattering angle and λ is the wavelength of the incident radiation] and with this a high resolution δ = 2π/*Q*_max_. If the nano-materials are small enough, structure models used for real-space fitting procedures may be large enough to cover the whole nano-crystallite or -particle (Teck *et al.*, 2017 ▸) and hence the area in which the correlation lengths are larger than the dimensions of the material and crystallite–crystallite, crystallite–particle or particle–particle interactions come into play. The software *DISCUS* (Proffen & Neder, 1997 ▸) follows this approach and models not just the internal structure of the nanomaterial but also the whole ensemble of crystallites and particles. Another approach, *e.g.* small-box refinements [see *e.g.* ch. 6 of Egami & Billinge (2003 ▸)], is to multiply an ideal infinite structure *G*(*r*) with an envelope function *f* (Howell *et al.*, 2006 ▸) which describes the shape and dimensions of the nanomaterial,
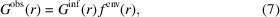
with *G*^obs^(*r*) the observed PDF, *G*^inf^(*r*) the PDF of the real infinite crystallite and *f*^env^(*r*) the envelope function.

### 1.1. Different envelope functions *f*^env^(*r*)

A good and widely used approximation for crystallite shapes is a sphere, which is mostly assumed in crystallite size determinations and close to the cube-shaped crystallite using the Scherrer equation with *k* = 0.94. Howell *et al.* (2006 ▸) used the assumption of spherical crystallites for their calculations and gave envelope functions for (i) a single crystallite as
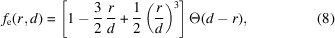
with *d* = 2*R* the crystallite diameter and Θ(*d* − *r*) the Heaviside step function [equation (6) of Howell *et al.* (2006 ▸)], and (ii) a distribution of such single spheres with a certain breadth (Howell *et al.*, 2006 ▸) as
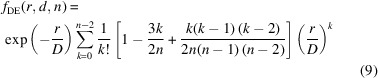
for 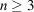.

A different approach followed by Beyer *et al.* (2022 ▸) starts from the viewpoint of Bragg scattering using pseudo-Voigt-type diffraction peaks, *i.e.* a linear combination of a Gaussian and a Lorentzian peak function, in which each function has a constant (crystallite size broadening) and a linear part (strain broadening). They calculated the influence of changes in the parameters of these functions on *G*(*r*) and thus on the envelope function. This resulted in the observation that such pseudo-Voigt functions are much better suited to reproducing atomic displacement parameters (ADPs) obtained from reciprocal-space refinements in total scattering experiments than the simple Gaussian parameters *Q*_damp_ and *Q*_broad_ used for example in *PDFgui* (Farrow *et al.*, 2007 ▸). However, this approach seems to be most probably limited to crystalline samples combined with high-resolution total scattering data. Furthermore, the determination of the ADPs depends strongly on the used wavelength: the longer the wavelength the smaller the influence of the ADPs on the atomic form factor.

Keeping the Scherrer equation in mind, a crystallite is the average volume of a sample in which the Bragg equation is fulfilled and gives rise to diffraction. Consequently, the crystallite size is the apparent average volume measure of the diffraction when dealing with reciprocal-space data. When the pair distribution function is considered, all contributions to the scattering are included and this also means diffuse contributions outside the Bragg reflections. Additionally, symmetry considerations do not play any role. In this case it is more reasonable to call such a volume an apparent average (pair) correlation volume and the diameter of a spherical crystallite obtaining the same volume the apparent average crystallite size. The word ‘apparent’ is used intentionally, because the peak broadening effects are not only caused by the sample’s crystallite size but also depend on the ‘visibility’ of the crystallite size in the specific diffraction experiment, particularly on the X-ray wavelength used.

### 1.2. ACS lower and upper limits

Besides all instrumental effects, nature sets the lower limit for diffraction of solid materials to a minimum number of atoms allowing us to distinguish between inner (bulk) and outer (surface) atoms. Assuming a close-packed arrangement as the most condensed one, this is realized in a cubic or hexagonal packing of 13 atoms, one inner and 12 outer atoms. The size limit of such a quantum-crystallite is in the range of ∼0.8 nm. Successful detection of diffraction from such a material is ultimately a question of measurement time.

**Figure 2:**
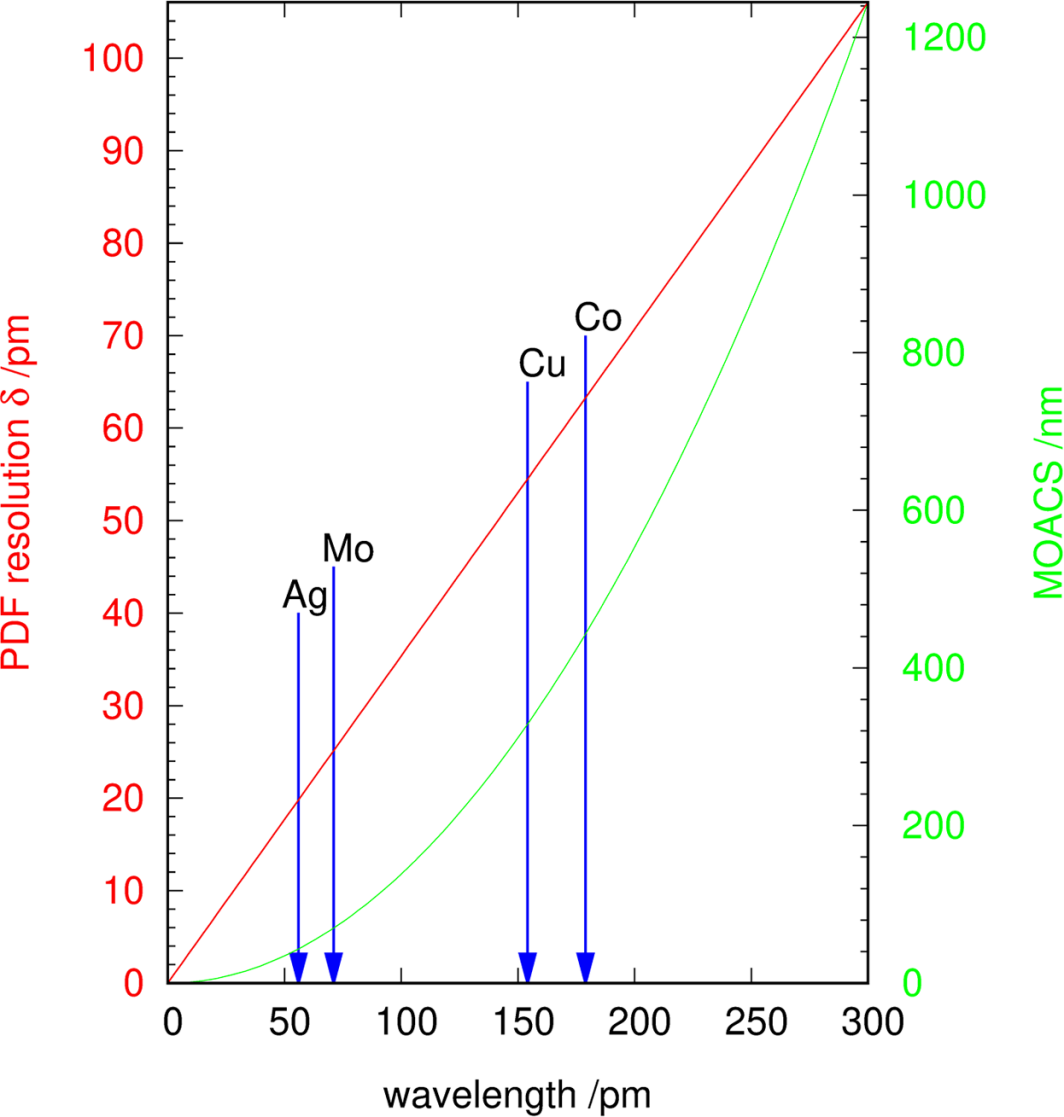
MOACS versus wavelength according to equation (10) using the natural spectral width of Cu radiation Δλ = 3.615 × 10^−5^ nm and PDF resolution δ assuming a maximum scattering angle 2θ of 135°.

One point rarely discussed is the existence of an upper limit for the determination of the ACS, stemming from the properties of the X-ray source used in the scattering experiments: the ACS must be smaller than the maximum coherence length of the radiation used. Thus, the ‘maximum observable ACS’ (MOACS) is an intrinsic property of the diffractometer used and can be derived (Rafaja *et al.*, 2000 ▸) as
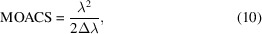
with λ the wavelength used and Δλ the natural spectral width of the source. Therefore, the MOACS increases quadratically with the used X-ray wavelength (Fig. 2 ▸), whereas the resolution in the PDF gets worse (Fig. 2 ▸). In-house standard X-ray diffraction with Cu radiation gives a MOACS of ∼280 nm and, in the best case, a PDF resolution of ∼50 pm (assuming a maximum scattering angle 2θ of 135°). Additionally, the influence of the ACS on the profile breadth depends on the scattering angle. Guinier [p. 146 of Guinier (1963 ▸)] gives the relation ACS ≃ 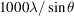 as an estimator, resulting in a MOACS of 180 nm at a 90° scattering angle 2θ for Cu radiation. The link between the intrinsic MOACS and the determined ACS, *e.g.* from Rietveld refinements, is only obvious in everyday laboratory work when data from different diffractometers (in-house and large-facility synchrotron data for example) are compared. The assumption that the ACS is a material property and as such a constant in all measurements is true from the viewpoint of the material but not from the point of view of the measurement, in which the ‘visibility’ could drastically be reduced.

To draw an intermediate conclusion: the longer the wavelength, the more the focus of the diffraction shifts to large-scale effects and vice versa. Another important consequence is that, if the average crystallite size of the sample is larger than the MOACS of a diffraction experiment, only the MOACS can be observed and in this way determined.

These correlations were the starting point for the procedure presented here. The *G*(*r*) calculated from in-house Cu (or longer wavelength) diffraction data naturally does not have a sufficient resolution for structure determinations. But if the long-range information is considered it should contain details about the crystallite size and the respective crystallite size distribution in its envelope. By this means, the ACS could be evaluated without dealing with the pitfalls of the Scherrer equation. A problem to be solved in this respect is that the observed *G*(*r*) has such a poor resolution that the corresponding structure cannot be refined and therefore it is practically impossible to use equation (7). A procedure named EnvACS to overcome this obstacle is presented in the following, meaning that no structural information is necessary. Subsequently it is shown that this method works on computed data, enabling the retrieval of input values for spherical crystallites. Our own standard reference material diffraction data, as well as such data generously provided by colleagues, measured with different wavelengths and diffraction geometries, were used to determine the MOACS and to show the influence of the measurement properties on the data and the ACS. The results provided by the EnvACS procedure are compared with those of Rietveld refinements of the diffraction data of commercial TiO_2_ with defined average particle sizes. Finally, the obtained distributions are compared with published distributions of core–shell nanomaterials.

## 2. Experimental

### 2.1. Diffraction data

Our own diffraction data from standard reference material (SRM) LaB_6_ were collected using diffractometers with different configurations:

(i) A Stoe Stadi-MP diffractometer, equipped with an Mo tube and a primary Ge(111) monochromator, producing pure α_1_ radiation, and a Dectris Mythen detector. For transmission measurements the sample was prepared either in a glass capillary (Debye–Scherrer, DS) or in a flat sample holder (TR). The diffractometer can also be configured for reflection measurements in Bragg–Brentano (BB) geometry.

(ii) A Bruker D8 Discover (D8D) diffractometer equipped with a Cu tube and a LynxEye XE-T detector.

(iii) A Bruker D8 Advance (D8A) diffractometer equipped with a Cu tube, a primary Johannson monochromator and a LynxEye detector. Both Bruker diffractometers were operated in BB geometry.

(iv) Finally, a Panalytical X’Pert MPD diffractometer, equipped with a Cu tube, a Ni foil filter and an X’Celerator detector, also operated in BB geometry.

(v) Synchrotron data were collected at DESY PETRA III on the P02.1 station (within proposal I-20220093) using a wavelength of 20.7311 (1) pm.

We gratefully thank the following colleagues for providing LaB_6_ scattering data: Miriam Zobel, RWTH Aachen (Stadi-MP multidetector system with monochromated Ag radiation); Claudia Weidenthaler, MPI für Kohlenforschung Mühlheim (Anton Paar XRDynamic 500, Bragg–Brentano, Co radiation); and Bilal Gökce, University of Wuppertal [data from the Advanced Photon Source BM-33-C, wavelength 77.4975 (1) pm, data presented by Nadarajah *et al.* (2021 ▸)].

#### 2.1.1. Rietveld refinements

Rietveld refinements were carried out using the software *TOPAS* (Coelho, 2018 ▸; Bruker AXS, Germany) or *GSAS-II* (Toby & Von Dreele, 2013 ▸) The instrument profiles that were used for both programs were fitted to LaB_6_ standard measurements for the respective diffraction geometry using the determined MOACS as the crystallite size of the SRM.

### 2.2. Data treatment and PDF generation

**Figure 3:**
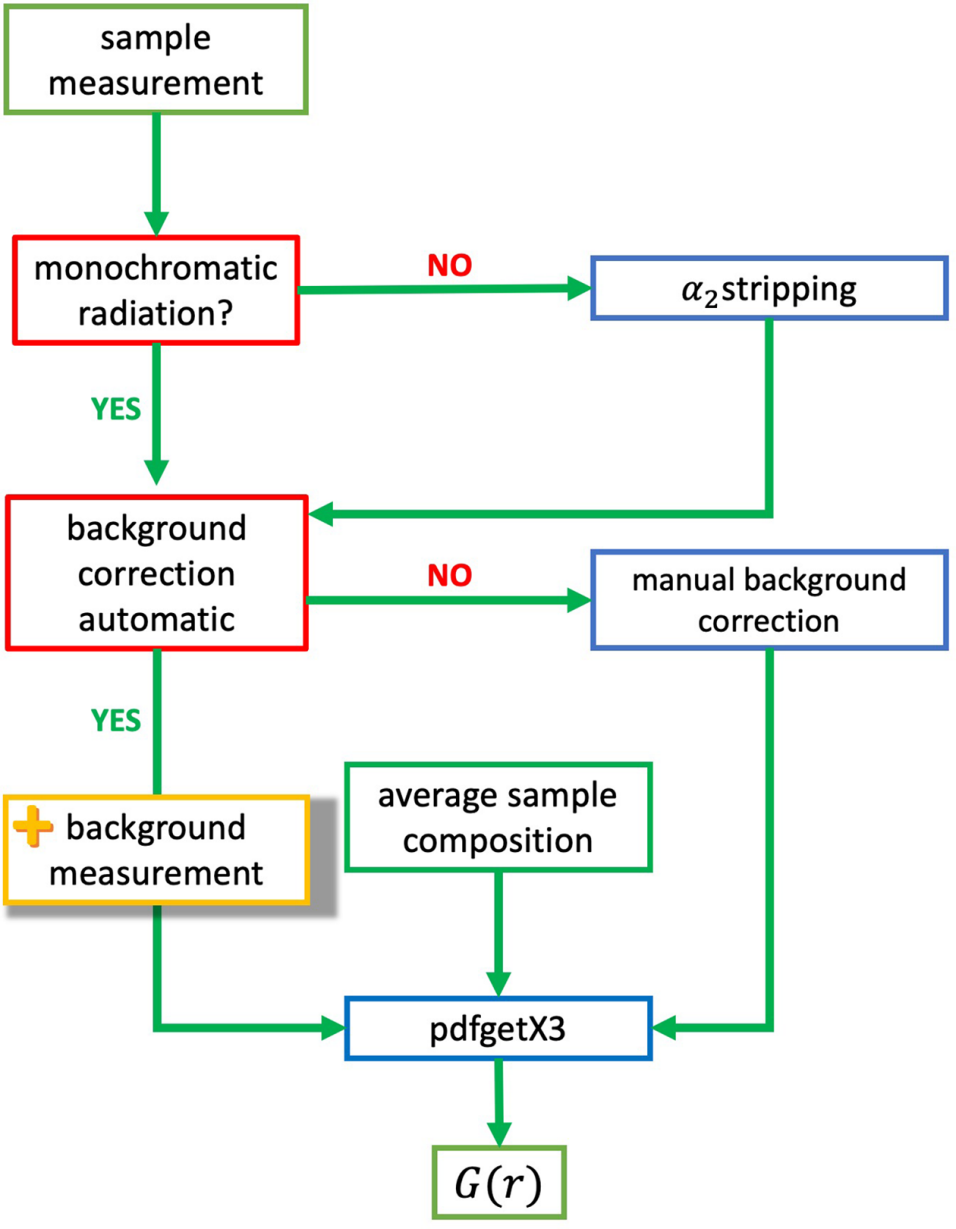
Data treatment for the calculation of *G*(*r*) using *PDFgetX3* for an EnvACS analysis.

To calculate *G*(*r*) from measured diffraction data the software *pdfGetX3* (Juhás *et al.*, 2013 ▸) was used. The pre-treatment of the diffraction data is shown schematically in Fig. 3 ▸. If non-monochromatic radiation was used, an α_2_-stripping based on the method published by Rachinger (1948 ▸) was carried out. If no background measurement was available, a manual correction was performed. This was mainly the case if the data were collected in BB geometry, for which a constant linear background contribution plus a 1/*X* contribution due to air scattering at low angles could be expected. If Rietveld refinements were carried out the calculated background curves from the final refinements were taken. Note that α_2_-stripping would only be an acceptable tool for microcrystalline (Gesing *et al.*, 2022 ▸) samples when using the data for an EnvACS analysis. This procedure should never be used for small nanocrystalline or quantum-crystalline samples, or for structure refinements at all, as it strongly reduces the data quality.

### 2.3. Approximation of the envelope by extracting the maxima

*G*(*r*) contains information about the internal atomic arrangement of the samples’ crystallites as well as information about their size distribution, and with this their average size and the crystallite–crystallite contacts. The experimentally observed *G*^obs^(*r*) can be described as a product of *G*^inf^(*r*) and *f*^env^(*r*) as given in equation (7). *G*^inf^(*r*) is the reduced radial distribution function of an infinite ideal crystal and *f*^env^(*r*) is the envelope function. The envelope function describes the form and distribution of the crystallites (Howell *et al.*, 2006 ▸; Neder & Korsunskiy, 2005 ▸). The EnvACS approach is to approximate *G*^obs^(*r*) by distinct points and thus finally to extract the experimental *f*^env^(*r*) without using *G*^inf^(*r*). The distinct points of *G*^obs^(*r*) are the absolute maxima in a certain *r* range. To enable this, the following procedure and definitions were used:

(i) The observed *G*^obs^(*r*) is normalized using its absolute maximum value max(*G*^obs^(*r*)), 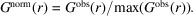


(ii) *G*^norm^(*r*) is divided into *N* intervals between *r*_start_ and *r*_end_ designated with *i*_*n*_ and a length *l*.

(iii) Let max(*i*_*n*_) be the maximum value of *G*^norm^(*r*) in the interval *i*_*n*_. Accordingly, the corresponding *r* value for this maximum is 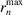.

(iv) Finally, *G*^norm^(*r*) is approximated by the sequence 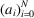 with *a*_*i*_ being the tuple 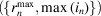.

Thus equation (7) can now be written in terms of this approximation: 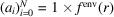. This approximation enables the fitting of the parameters of *f*^env^(*r*) against the values contained in the series 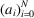. This procedure was implemented in a Python script, which carries out all the steps described above and fits the envelope parameters to the series of 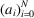 values.

## 3. Results and discussion

### 3.1. Proof of concept using synthetic data

To show the validity of the procedure, theoretical *G*(*r*) of LaB_6_ up to 20, 40, 60 and 120 nm were calculated using *PDFgui* (Farrow *et al.*, 2007 ▸) and an X-ray wavelength of 100 pm. The spherical crystallite diameter (SCD) function implemented in *PDFGui* was varied between 1 and 125 nm. The envelope functions of the calculated *G*(*r*) were extracted and fitted using three models:

(i) The model of Beyer *et al.* (2022 ▸) without consideration of strain contributions (denoted Beyer *et al.*).

(ii) A model based on that of Howell *et al.* (2006 ▸) using a mixture of a single spherical crystallite (smallest size distribution) and a distribution with *n* = 3 (biggest size distribution) using a mixing parameter [denoted Howell *et al.* (mix)].

(iii) A distribution based on that of Howell *et al.* (2006 ▸) in which, besides *D*, *n* was also determined [denoted Howell *et al.*(*n*/*D*)].

**Figure 4:**
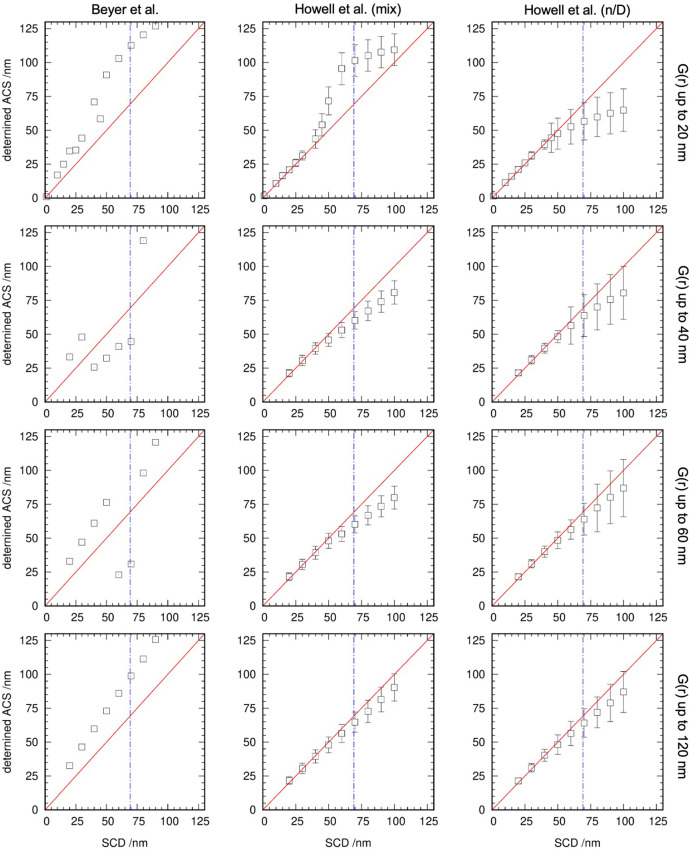
ACS determined using the three different models named in the top row from synthetic *G*(*r*) up to 20, 40, 60 and 120 nm. The red solid line is the 1:1 line and the blue dot–dashed line is half the MOACS.

The best fitting results, as can be seen in Fig. 4 ▸, were obtained using model (iii) [Howell *et al.* (*n*/*D*)]. Additionally, it can be seen that the deviations from the expected values and the errors in the obtained ACS become smaller using a longer *r* range in the calculation of *G*(*r*).

### 3.2. Determination of the ACS of LaB_6_ SRM using different wavelengths and diffraction geometries

**Figure 5:**
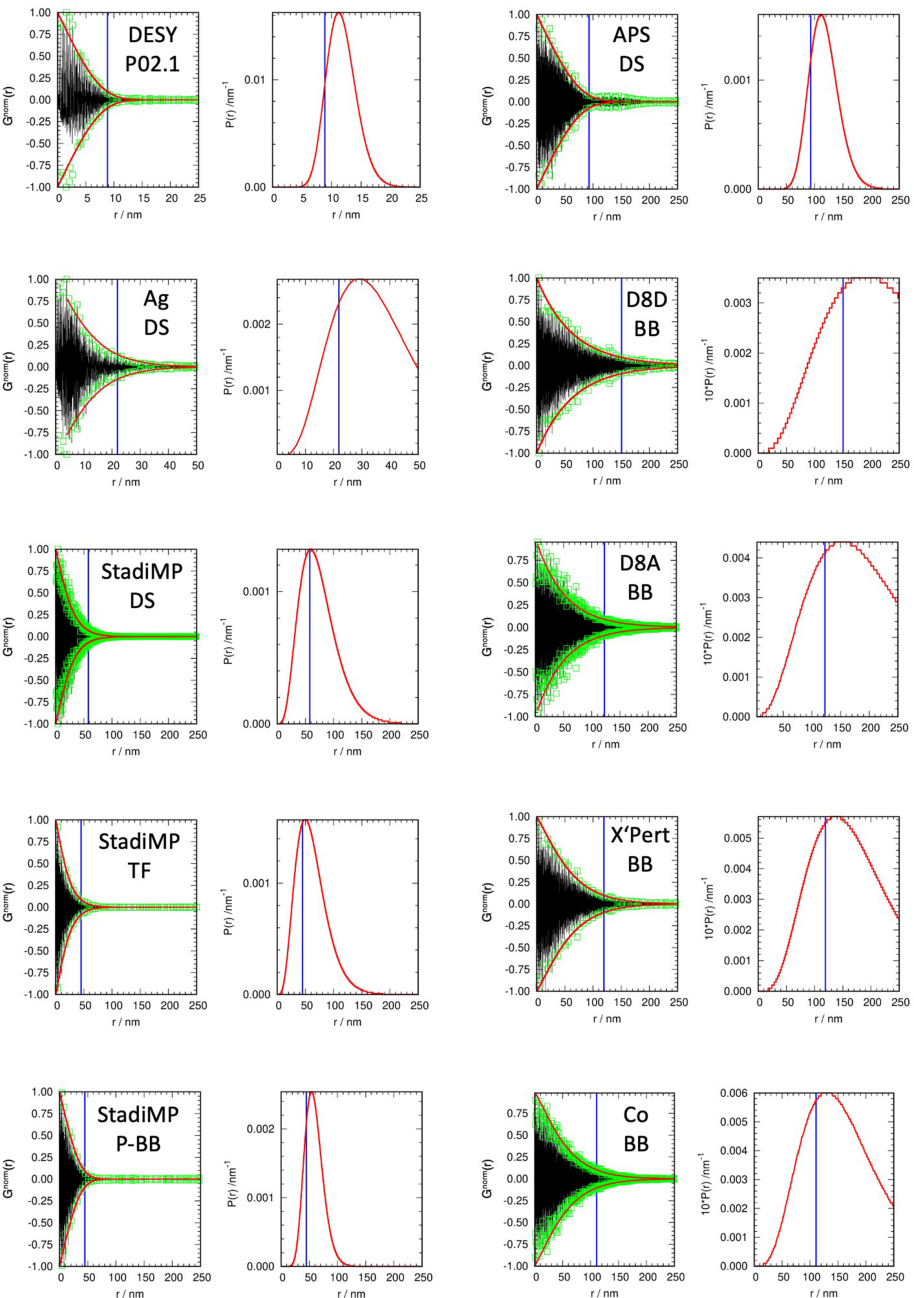
(First and third columns) Determined *G*^norm^(*r*) (black), *a*_*i*_ (green squares) and envelope function (red curves), and (second and fourth columns) distribution function (red) and MOACS calculated using the coherence length value of Rafaja *et al.* (2004 ▸) (vertical blue line), for all wavelengths and geometries of the LaB_6_ SRM measurements.

LaB_6_ is the most commonly used SRM [National Institute of Standards and Technology (NIST), Maryland, USA; SRM660c] in X-ray powder diffraction. One can safely assume that the ACS and strain broadening do not play any role in diffraction experiments using this material (Black *et al.*, 2020 ▸). Besides our own collected diffraction data, several colleagues provided standard measurements on additional machines, which are gratefully acknowledged. Using the EnvACS procedure, the ACS of LaB_6_ was determined (Fig. 5 ▸). Data sources and details and the results of the calculations are collected in Table 1 ▸.

**Figure 6:**
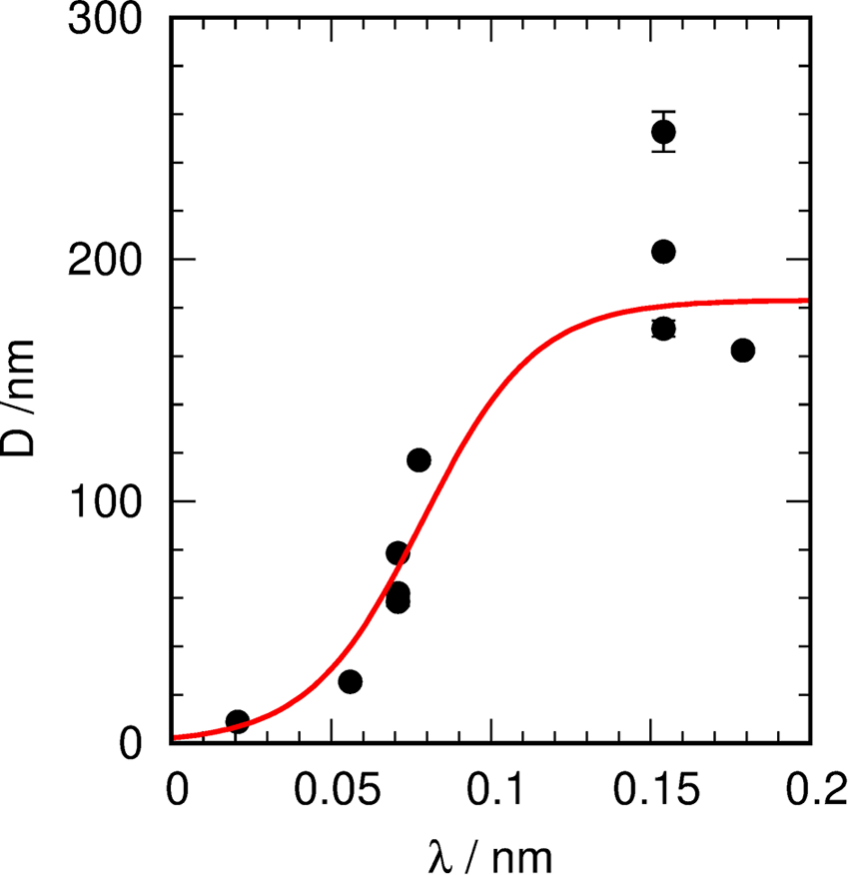
The *D* parameters from the envelope fitting collected for all wavelengths and geometries considered. The red line shows the fit using a logistic function.

The results show that the MOACS depends, as expected, on the used wavelength and diffraction geometry. Fitting *D* with a logistic function (see Fig. 6 ▸),
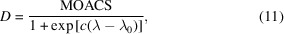
results in MOACS = 184 (13) nm, λ_0_ = 0.078 (5) nm and *c* = 56 (12) nm^−1^. The results are consistent in themselves but also show a strong influence of the diffraction geometry for Cu radiation. There are in principle three effects which mainly influence the MOACS:

(i) Using a monochromator will increase the MOACS due to reduction of the coherence length of the radiation during monochromatization.

(ii) The crystallinity of the sample itself could change the coherence length. In particular, if the crystallites are bigger than the MOACS, they act themselves like monochromators, thus influencing the coherence length. The better crystallinity the samples have, the stronger could this effect be. Nevertheless, such an effect can be excluded while always using the same standard sample.

(iii) Data manipulation, like α_2_-stripping, additionally influences the MOACS as the mathematical treatment influences not only the α_2_ reflections but also the remaining α_1_. This is at first glance apparent not as a change in the coherence length but as a narrowing of the α_1_ reflections themselves, resulting in a bigger MOACS.

Further details of these effects are given by Rafaja *et al.* (2004 ▸, 2000 ▸) and Guinier (1963 ▸). However, all beam-forming elements in the radiation path (including the sample) could affect the machine’s apparent coherence length, and the use of a standard reference material and this procedure allows the determination of this parameter of the diffractometer. With regard to the remarks of Guinier (see *Introduction*[Sec sec1]), these results confirm his estimation of an upper boundary of ∼200 nm for the determination of the ACS ‘*even with the best instruments*’ [p. 146–147 of Guinier (1963 ▸)].

### 3.3. ACS of commercial TiO_2_

**Figure 7:**
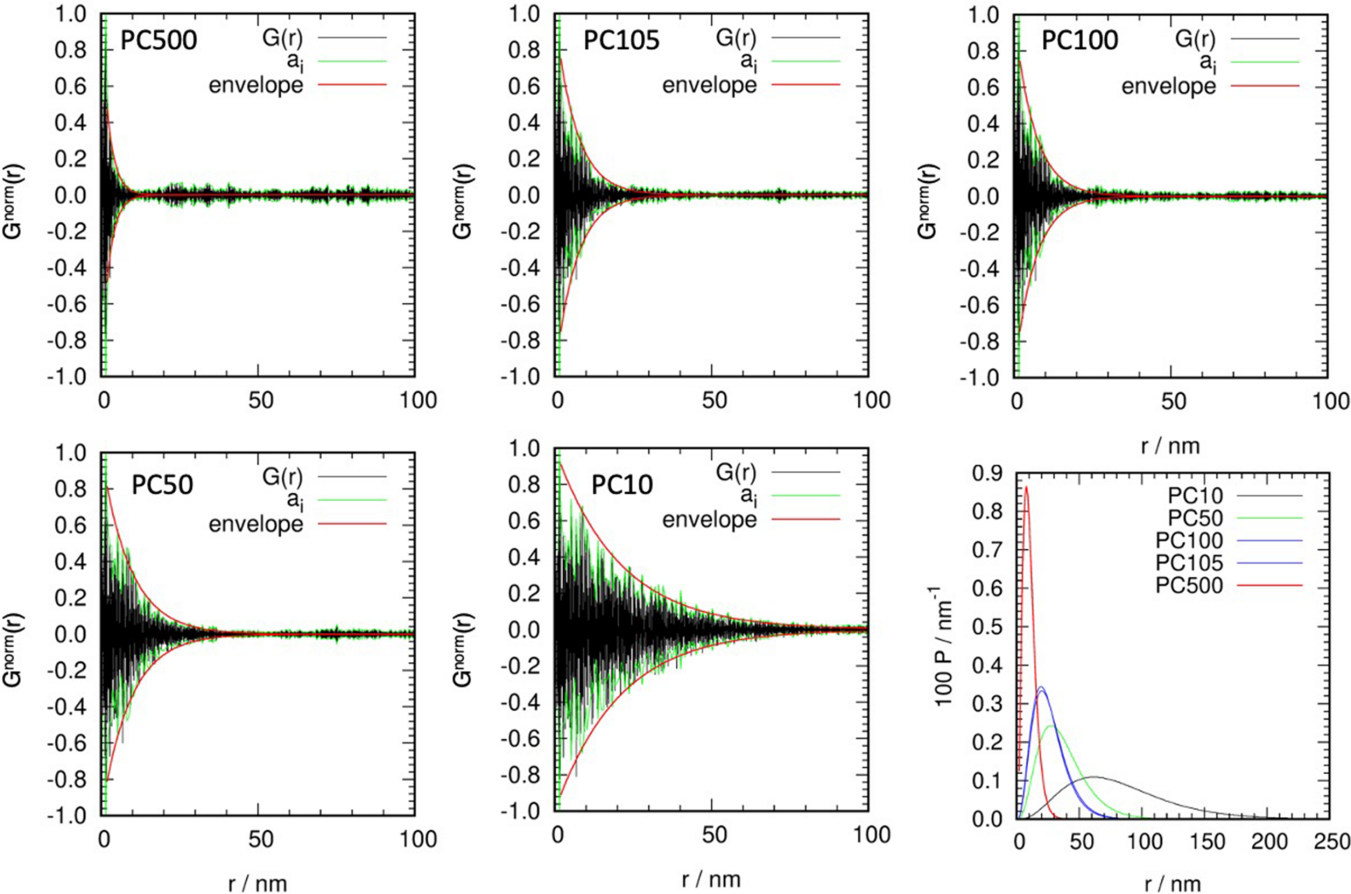
Determined *G*^norm^(*r*) (black), *a*_*i*_ (green) and envelope function (red) for the five TiO_2_ materials. The bottom right panel gives the evaluated distribution functions.

As a reference test case commercial TiO_2_ samples (pure anatase) were used, which were manufactured with different average particle sizes. The ACS values were determined using Rietveld refinements and the EnvACS procedure. The final ACS values and the reference values are collected in Table 2 ▸, and *G*(*r*), *a*_*i*_ and the fitted envelopes of the five samples and the obtained distribution functions are shown in Fig. 7 ▸.

**Figure 8:**
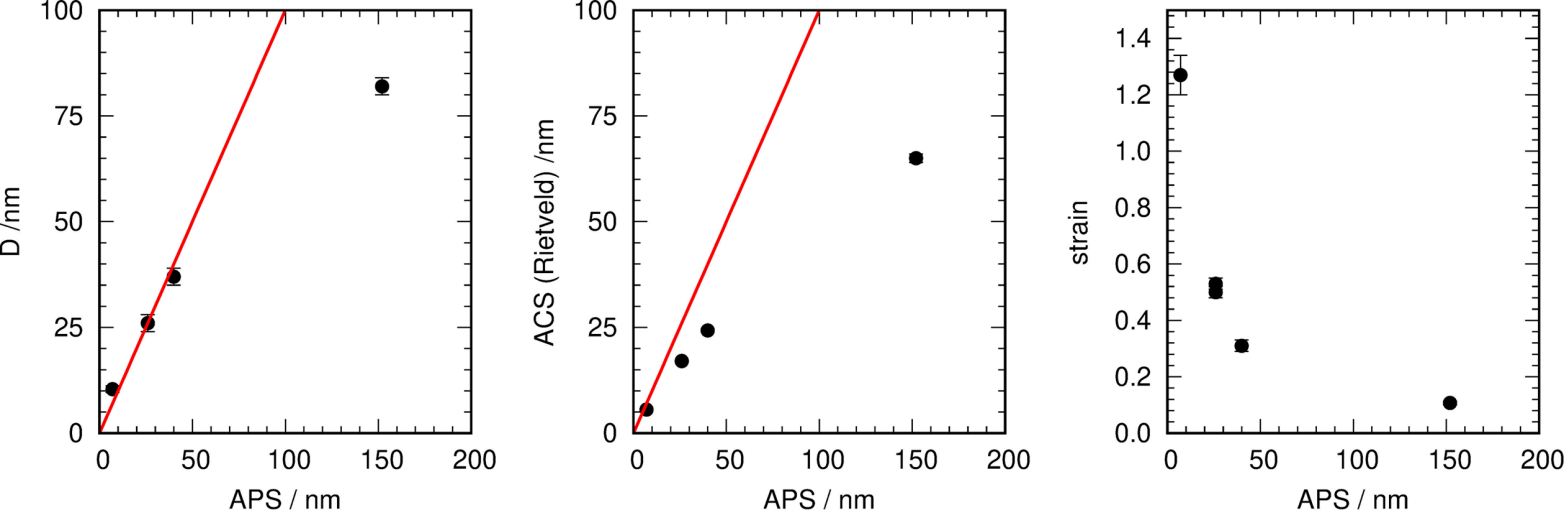
(Left) The obtained *D* values from the fitted envelopes of commercial anatase; (middle) the ACS and (right) the strain from Rietveld refinements plotted versus the manufacturer’s APS. The red line is the 1:1 ratio line.

Correlations between the manufacturer’s APS and the *D* value obtained from the envelope fitting, as well as the ACS and the strain obtained from Rietveld refinements, are shown in Fig. 8 ▸. The ACS determined from Rietveld refinements is always below the manufacturer’s average particle size. The determined strain decreases with increasing crystallite or particle size, showing the increasing bulk contribution to the crystallite morphology (Gesing *et al.*, 2022 ▸). The *D* parameter of the fitted distribution agrees well with the average particle size for all samples except PC 10, where *D* is approximately half as large as the average particle size, which could hint that these large particles do in fact consist of two crystallites. It is noteworthy that the ACS obtained from the Rietveld refinements is quite close to the maximum position of the distribution functions [which could be calculated by 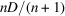]. This shows that the strong deviation of the ACS obtained from the Rietveld refinements from the 1:1 line is caused by the distribution of crystallite sizes in the samples [compare Scardi & Leoni (2006 ▸)]. Additionally, the smaller the ACS is, the smaller is the influence of the distribution, because the distribution’s σ must be small. The data show an increase in the deviation with increasing ACS, while at the same time the strain is reduced drastically and thus can be excluded as a reason for this behavior (Fig. 8 ▸).

### 3.4. Crystallite versus particle size distributions

**Figure 9:**
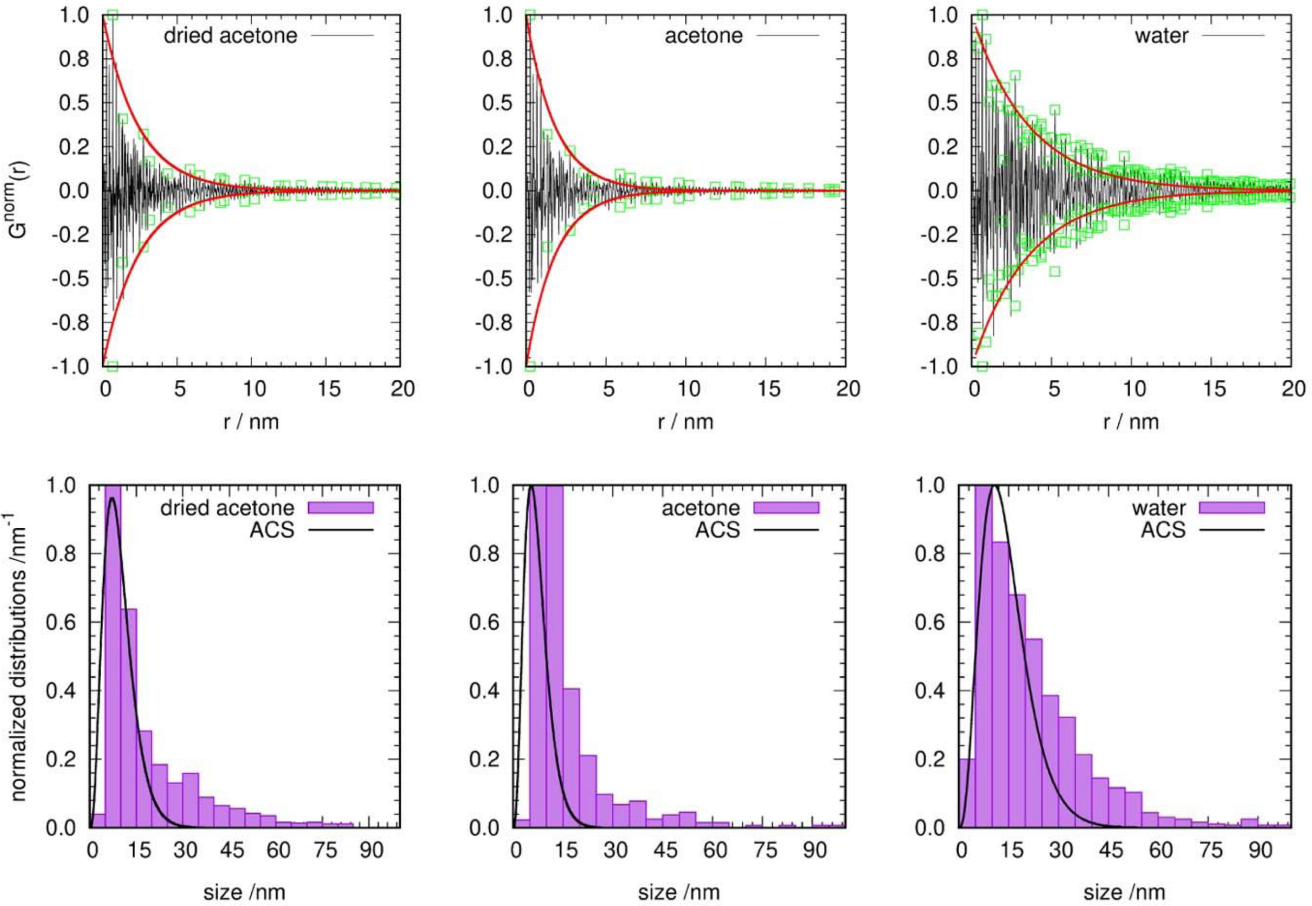
(Upper row) Normalized *G*^norm^(*r*) (black), determined maxima (green squares) and fitted envelope (red), and (lower row) normalized particle size distributions (purple bars, taken from the reference) and the determined ACS distributions (black lines, calculated by the new procedure using data provided by B. Gökce, normalized), for the three different syntheses examined by Khairani *et al.* (2023 ▸).

For the Fe–Ni alloy core–shell nanoparticles investigated by Bilal Gökce and co-authors (Khairani *et al.*, 2023 ▸), the particle size distributions (PSDs) obtained using transmission electron microscopy were compared with sizes recalculated from synchrotron scattering data measured for three different syntheses (Fig. 9 ▸), which were kindly provided for our investigation. The synchrotron data were measured using a wavelength of 77.4975 (1) pm (beamline 33-BM-C at the Advanced Photon Source, Argonne, Illinois, USA) and data were collected in either glass or Kapton sample containers for the purpose of Rietveld refinements. Due to the lack of a sample container background measurement, the data were manually corrected and only the glass container data were used, because the container contributions seemed to be not as strong as those from the Kapton containers. The final ACS distributions are shown in Fig. 9 ▸ as black lines. For the ‘water’ synthesis the maxima of the PSD and the ACS distribution fit quite well, although for sizes above 30 nm the differences become quite large. This discrepancy is caused by the difference in the shape of the two distributions – the distribution used is not the log–normal distribution generally used for the PSD and is not able to be skewed to the same degree as a log–normal distribution. The same effects (good fitting of the maxima, stronger deviation for larger sizes) can be observed for the ‘dried acetone’ sample. In the case of the ‘acetone’ sample the maximum of the ACS distribution clearly seems to underestimate the real distribution. Ultimately, it can be concluded that the overall agreement of the ACS distribution is quite good, considering the rough background correction, the core–shell character of the particles themselves and their variable chemical composition, which could also not be considered in the calculation of *G*(*r*) from the scattering data.

## 4. Summary and conclusion

A new approach to determine the apparent average crystallite size from in-house diffraction data has been presented. In this approach the diffraction data are transformed to *G*^norm^(*r*) which is then approximated by its maximum values in certain *r* ranges. The maximum series is fitted by a distribution function for spherical particles. This approach was tested using synthetic data and three different distribution functions; the best working approach by Howell *et al.* (2006 ▸) was used for further evaluations.

Using the SRM LaB_6_ diffraction data measured on a variety of diffraction geometries and at different wavelengths showed that the maximal observable apparent average crystallite size depends on the wavelength, with an upper limit around approximately 200 nm for long wavelengths. However, this approach easily allows the determination of the individual machine MOACS.

Two test cases have been considered: commercial TiO_2_ anatase and Fe–Ni alloy nanoparticles. In the first case the new method gives ACS values which correspond very well to the ones given by the manufacturer. Respective values determined by Rietveld refinements deviate more strongly from the given values the larger the ACS gets. This is caused by the increasing influence of the distribution function, an effect already noticed by Scardi & Leoni (2006 ▸). In the second case [data provided by Khairani *et al.* (2023 ▸)] the new method complies quite well with the observed average particle size distributions but shows shortcomings because of the different nature of the used and observed distribution functions. However, in principle it is easily possible to use different distribution functions adapted to the respective geometry of the sample (Usher *et al.*, 2018 ▸; Leonardi *et al.*, 2022 ▸).

The biggest advantage of the method is that no profile fitting or similar techniques of reciprocal-space diffraction data are necessary, so all pitfalls of the Scherrer equation are avoided. Secondly, it allows the determination of the average crystallite distribution. Finally, this method could also be used to determine the exact MOACS of an instrument, which could then be used to parameterize exactly the instrumental function for any Rietveld approach.

## Figures and Tables

**Table 1:** Data sources and details Used geometries: DS – Debye–Scherrer, TR – transmission (flat sample holder), BB – Bragg–Brentano, P-BB – pseudo-BB. *D* is the ACS and σ the characteristic width of the distribution obtained by Howell *et al.* (2006 ▸).

Source	Device	Wavelength/pm	Geometry	Monochromatic radiation?	*D*/nm	σ/nm
DESY (our own data)	P02.1	20.7311 (1)	DS	Yes	8.75 (22)	2.76 (1)
M. Zobel	StadiMP	55.94218 (8)	DS	Yes	25 (2)	2.54 (1)
Own	StadiMP	70.93171 (4)	DS	Yes	78.6 (6)	32.08 (4)
Own	StadiMP	70.93171 (4)	TR	Yes	62.0 (6)	27.74 (5)
Own	StadiMP	70.93171 (4)	P-BB	Yes	58 (2)	16.24 (4)
Advanced Photon Source (Gökce)	BM-33-C	77.5	DS	Yes	117 (2)	25.51 (1)
Own	D8 Advance	154.05929 (5)	BB	Yes	203 (1)	101.6 (2)
Own	D8 Discover	154.05929 (5)	BB	No	253 (8)	126 (1)
Own	X’pert MPD	154.05929 (5)	BB	No	171 (3)	76.6 (4)
Weidenthaler	Anton Paar XRDynmic 500	178.901 (1)	BB	No	162 (1)	72.61 (8)

**Table 2:** Results from Rietveld refinements (ACS and strain) and EnvACS (ACS, distribution width) for TiO_2_ materials (APS is the average particle size as given by the manufacturer)

Properties		Rietveld refinements		EnvACS	
Sample	APS/nm	ACS/nm	Strain	*D*/nm	σ/nm
PC 500	7	5.57 (8)	1.27 (7)	10.4 (8)	1.3 (1)
PC 105	26	17.0 (2)	0.53 (2)	26 (2)	3.35 (15)
PC 100	26	17.1 (2)	0.50 (2)	26 (2)	3.25 (15)
PC 50	40	24.3 (3)	0.31 (2)	37 (2)	4.6 (2)
PC 10	152	65 (1)	0.107 (6)	82 (2)	10.3 (2)
